# Keratan sulfate expression in microglia is diminished in the spinal cord in experimental autoimmune neuritis

**DOI:** 10.1038/cddis.2013.479

**Published:** 2013-12-05

**Authors:** H Matsui, T Ohgomori, T Natori, K Miyamoto, S Kusunoki, K Sakamoto, N Ishiguro, S Imagama, K Kadomatsu

**Affiliations:** 1 Department of Biochemistry, Nagoya University Graduate School of Medicine, Nagoya 466-8550, Japan; 2 Department of Orthopedics, Nagoya University Graduate School of Medicine, Nagoya 466-8550, Japan; 3 Department of Health and Nutrition, Yamanashi Gakuin University, Kofu 400-8575, Japan; 4 Department of Neurology, Kinki University School of Medicine, Sayama, Osaka 589-8511, Japan

**Keywords:** experimental autoimmune neuritis, keratan sulfate, microglia

## Abstract

Experimental autoimmune neuritis (EAN) is an animal model of Guillain–Barré syndrome, an inflammatory demyelination disease of the peripheral nervous system. Although this disease has been extensively studied on peripheral nerves, the pathology of the central nervous system has not been fully understood. Previous studies demonstrate that expression of keratan sulfate (KS), the sugar chain of proteoglycan, is associated with activated microglia/macrophages accumulated after neuronal injuries. Unexpectedly, we found here that KS is rather diminished in rat EAN. KS was restrictively expressed in microglia in the spinal cord of normal rats. KS was positive in 50% microglia in the ventral horn and 20% in the dorsal horn. In EAN, microglia increased in number and expressed the activation marker CD68, but KS expression was abolished. Concomitantly, pro-inflammatory cytokines, i.e., interferon (IFN)-*γ*, interleukin (IL)-1*β*, and tumor necrosis factor (TNF)-*α*, were increased in the spinal cord of EAN rats, whereas anti-inflammatory cytokines, such as IL-4 and IL-10, were decreased. In addition, silencing of KSGal6ST attenuated KS expression on the primary cultured microglia and upregulated expression of some activation markers (TNF-*α*, IL-1*β*, and iNOS) under the stimulation with lipopolysaccharide and IFN-*γ*. This study demonstrates for the first time a close association of EAN and disappearance of KS on microglia. KS expression could be a useful marker to evaluate the status of polyneuropathy.

Experimental autoimmune neuritis (EAN) is an animal model of human Guillain–Barré syndrome, and is a helper T-cell-mediated inflammatory demyelination disease in peripheral nerve that is affected by the immunization of P2 peptide.^1, 2, 3^ The pathology of peripheral nerves in EAN has been extensively studied; EAN involves disruption of blood nerve barrier, infiltration of T cells and macrophages, leakage of immunoglobulin, and regional demyelination.^4, 5, 6^ Although the central nervous system (CNS) has been poorly studied on EAN, some important findings on CNS have been reported. Microglia transiently increase in number and they express P2X4 receptor involved in allodynia and pain hypersensitivity in the dorsal horn of the spinal cord in EAN.^7, 8^ Moreover, infiltration of CD4-positive T cells is observed in the spinal cord and triggers apoptosis of motor neurons.^9^

Proteoglycans (PGs) have long sugar chains, so-called glycosaminoglycans, i.e., chondroitin sulfate, keratan sulfate (KS), dermatan sulfate, and heparan sulfate.^10, 11^ KS is a polymer of lactosamine, 3Gal*β*1–4GlcNAc*β*1, sulfated at the C6 of both hexose moieties. GlcNAc6ST-1 mediates sulfation of C6 position of GlcNAc residue, which is an essential step for KS biosynthesis.^12^ Therefore, GlcNAc6ST-1-deficient (GlcNAc6ST-1^−/−^) mice show loss of KS in the CNS.^13^ We and others reported^14, 15, 16^ that KS expression is enhanced in microglia/macrophages and the extracellular matrix after spinal cord injury (SCI). We found that GlcNAc6ST-1^−/−^ mice show better functional recovery after SCI.^15^ Ablation of KS with its degrading enzyme keratanase II also promotes functional recovery after SCI.^16^ Further *in vitro* studies revealed that KS in extracellular matrix has an inhibitory role in axonal regeneration/sprouting that is relevant to functional recovery with KS ablation after SCI.^16^ On the other hand, the biological significance of KS expressed in microglia/macrophages remains obscure.

In this study, we attempted to clarify the pathology of the CNS in EAN. We particularly focused on KS expression. Based on an association of KS expression and activated microglia/macrophages accumulated after neuronal injuries, we had speculated that KS expression might be upregulated in the spinal cord to exacerbate EAN. However, we found that KS was rather diminished in rat EAN. We have revealed for the first time the relationship of KS expression and EAN pathogenesis.

## Results

### The expression of KS is diminished in the spinal cord of EAN

**Figure 1:**
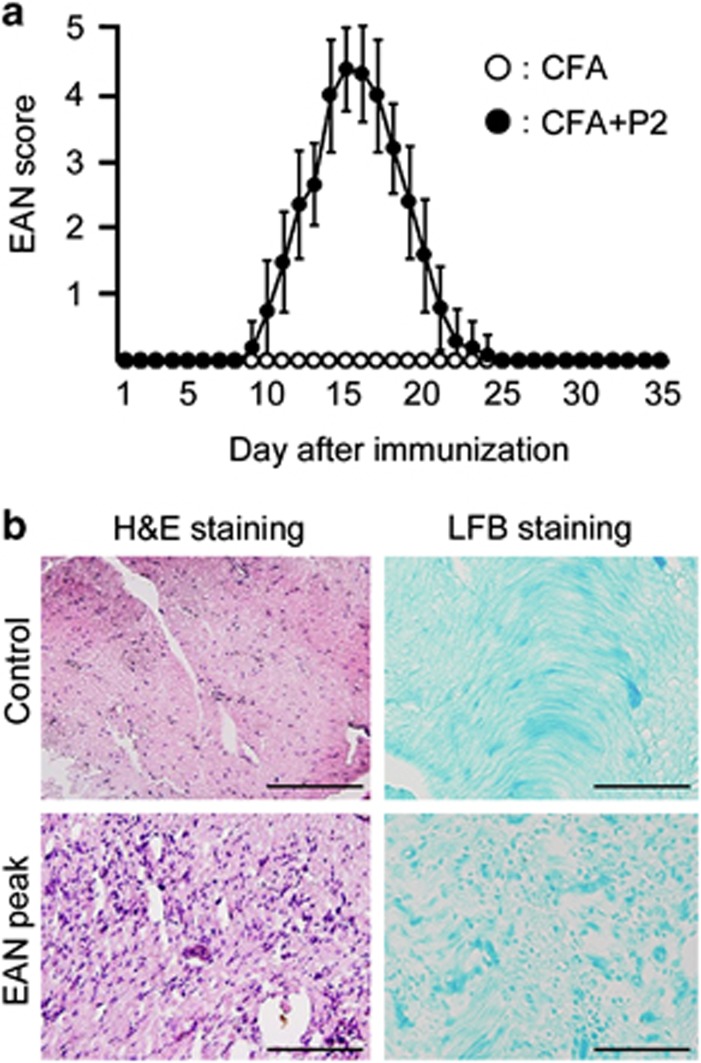
Clinical scores of EAN and pathological changes of SNs. (**a**) The clinical scores of control and EAN rats were evaluated every day after immunization of CFA and P2 peptide (*n*=15 per each groups). (**b**) The SNs of control and EAN rats were stained by H&E and LFB staining. Infiltration of inflammatory cells and demyelination were observed at the peak of EAN. Scale bars, 50 *μ*m

The clinical scores of control (complete Freund's adjuvant (CFA) alone: open circles) and EAN (closed circles) rats were shown in Figure 1a. The symptom of limp tail first appeared at 9 days after immunization and this point was defined as onset of EAN. The peak of symptoms was observed at 14–18 days and subsequently recovered at 25 days. No symptoms were observed in control rats. The histological analysis revealed that inflammatory cells strikingly infiltrated in the sciatic nerve (SN) at the peak of EAN (18 days after immunization) (Figure 1b, hematoxylin and eosin (H&E) staining). Demyelination of SNs was also observed in EAN (Figure 1b, Luxol Fast Blue (LFB) staining). On the other hand, infiltrated inflammatory cells and demyelination were not observed in control rats (Figure 1b).

**Figure 2:**
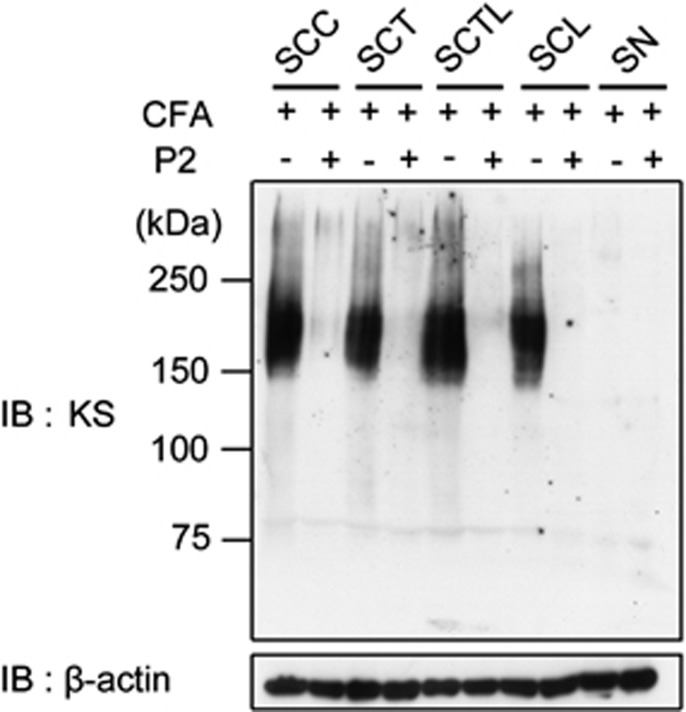
Expression of KS is abolished at the peak of EAN. KS expression was examined with the KS-specific antibody 5D4 for spinal cords and SNs of control and EAN rat. SCC, spinal cord cervical, SCT, spinal cord thoracic, SCTL, spinal cord thoraco–lumbar, SCL, spinal cord lumbar. *β*-actin was used as the internal loading control

The expression levels of KS in the spinal cord of control and EAN rats were analyzed by immunoblotting at the peak of EAN. The KS-specific antibody 5D4 was used in this experiment. Tissues were obtained from the cervical, thoracic, thoraco–lumbar and lumbar spinal cord, and SNs (Figure 2). KS was detected at the size of 150–250 kDa in all regions of the control spinal cords (Figure 2, lanes 1, 3, 5, and 7). As KS is a long sugar chain, KS-bearing PGs appear as smear bands as shown in Figure 2. Surprisingly, this reactivity disappeared in EAN (Figure 2, lanes 2, 4, 6, and 8). In contrast, KS could not be detected in SNs of both control and EAN rats (Figure 2, lanes 9 and 10).

### KS is specifically expressed in a subpopulation of microglia in normal spinal cord

**Figure 3:**
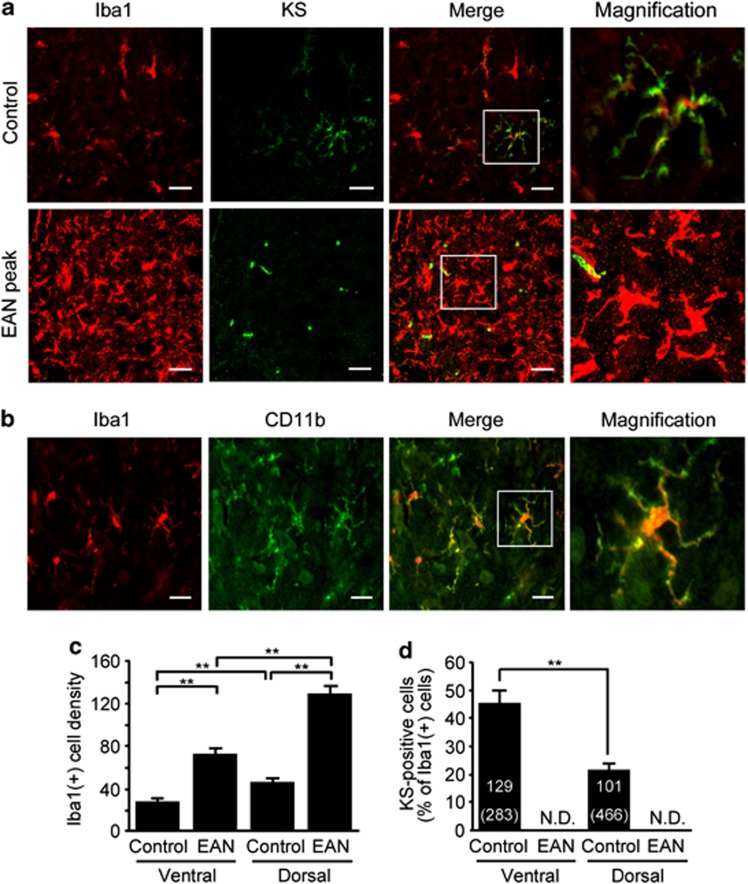
KS is specifically expressed on microglia/macrophages in the normal spinal cord. (**a**) The spinal cord frozen sections were from control or EAN rats and were stained with indicated antibodies. Scale bars, 20 *μ*m. The figure shows the representative images in the dorsal horn area of control and EAN rats. (**b**) Iba1-positive cells were completely merged with CD11b-positive cells in the normal spinal cords. Scale bars, 20 *μ*m. (**c**) The number of Iba1-positive cells was counted in the ventral and dorsal horns of the normal and EAN spinal cords (*n*=10). (**d**) The percentages of KS-positive cells were calculated in ventral and dorsal horns of the normal and EAN spinal cords. The numbers in parentheses indicate the number of microglia examined. Each column represents average±S.D. N.D., not detected. The *P*-value was calculated using Student's two-tailed *t*-test. **P<0.01

We next attempted to identify the source of KS expression. The KS-positive cells were a part of Iba1-positive microglia/macrophages in the spinal cord of normal rats (Figure 3a). Although the number of microglia/macrophage significantly increased at the peak of EAN, KS expression disappeared (Figure 3a). The morphology of microglia/macrophage was changed from ‘ramified' to ‘amoeboid' shape in the spinal cord of EAN, indicating that these microglia/macrophages were activated.^17^ In addition, we used CD11b (OX-42) as an additional marker for microglia/macrophages and found that expressions of Iba1 and CD11b were almost completely merged (Figure 3b). Therefore, all the Iba1-positive cells were CD11b positive. These data suggested that a subpopulation of microglia/macrophages expressed KS in normal spinal cord.

The number of Iba1-positive cells was increased in both ventral and dorsal horns at the peak of EAN (Figure 3c), consistent with a previous report.^7^ Iba1-positive microglia/macrophage predominantly increased in spinal cord dorsal horn. In normal spinal cords, the ratio of KS-positive microglia in Iba1-positive cells was significantly higher in the ventral horn (49±8.9%) than in dorsal horn (20±3.7%) (Figure 3d). KS expression was disappeared in both dorsal and ventral horns at the peak of EAN (Figure 3d).

### Spatio-temporal relationship of KS and microglia/macrophages activation markers

**Figure 4:**
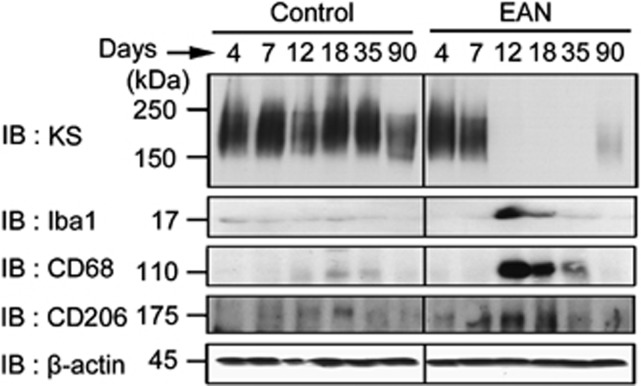
Reverse relationship of KS and microglia/macrophages activation markers. Expression of KS, Iba1, CD68, and CD206 was examined at 4, 7, 12, 18, 35, and 90 days after the immunization. *β*-actin was used as the internal loading control

We next examined the relationship between KS expression and microglia/macrophages activation in the context of the progression of EAN. KS could be detected in the spinal cord in pre-onset phase (4–7 days after immunization), and disappeared at onset (12 days), peak (18 days), and remission (35 days) phases (Figure 4, top panel). This reactivity was partially recovered at 90 days after immunization (Figure 4, top panel, lane 12). The expression level of the microglia/macrophage marker Iba1 significantly increased at 12 and 18 days, showing a mirror image of KS expression (Figure 4, second panel). It is known that activated microglia has two phenotypes, i.e., ‘classically activated' microglia (M1) and ‘alternatively activated' microglia (M2).^18, 19^ In general, M1 microglia/macrophages exert pro-inflammatory functions, whereas M2 microglia/macrophages show anti-inflammatory functions. Activation markers detected by antibodies available for rat microglia include CD68 and CD206. CD68 is usually induced upon activation and may be positive for both M1 and M2 microglia, whereas CD206 exclusively represent a subset of M2 microglia. We found that both CD68 and CD206 were strongly expressed during EAN, showing a mirror image of KS expression (Figure 4, third and fourth panels).

**Figure 5:**
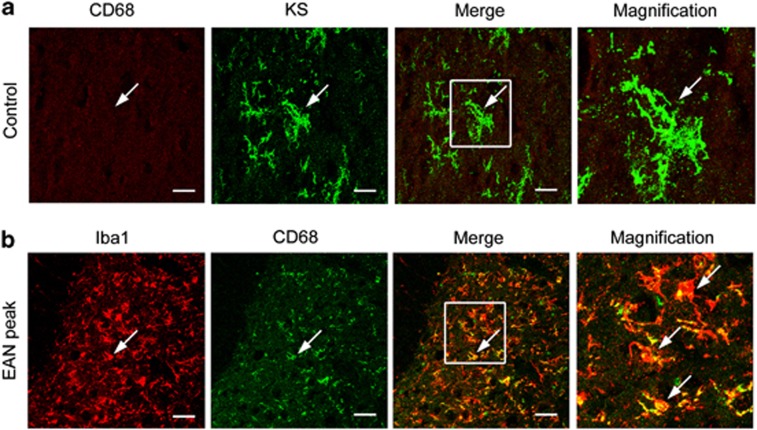
The microglia/macrophages activation marker CD68 is expressed in EAN. (**a**) Expression of CD68 and KS was examined for control rat spinal cord. (**b**) Expression of Iba1 and CD68 was examined for EAN rat spinal cord. Scale bars, 20 *μ*m. The figure shows the representative images in the dorsal horn area of control and EAN rats

**Figure 6:**
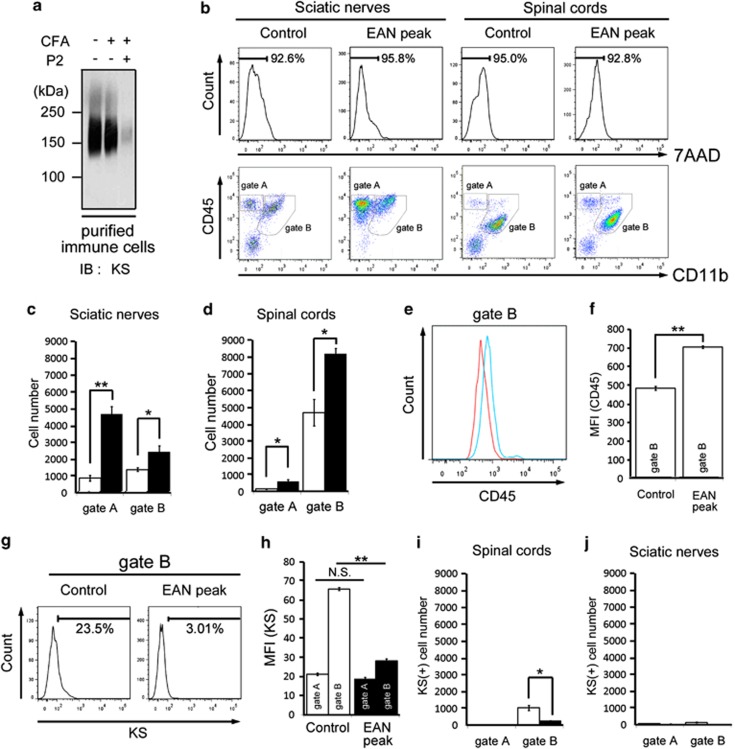
Flow cytometric analysis of immune cells from the spinal cord and SNs separated with Percoll centrifugation. (**a**) Total cell lysates of spinal cord immune cells enriched with Percoll centrifugation were subjected to immunoblotting with the anti-KS antibody. (**b**) Immune cells purified from SNs and spinal cords were subjected to flow cytometry analysis. Representative results are shown (*n*=3). Gate A corresponds to lymphocytes, gate B corresponds to microglia/macrophages (**c**). The number of lymphocytes and macrophages were counted in normal and EAN SNs. (**d**) The number of lymphocytes and microglia/macrophages were counted in normal and EAN spinal cords. (**e**) Expression of CD45 was analyzed by flow cytometry. Red line and blue line correspond to control and EAN peak, respectively. (**f**) Mean fluorescent intensity of CD45 was calculated in microglia/macrophages (gate B). (**g**) KS expression in the microglia/macrophages (gate B) in the spinal cords was measured. (**h**) Mean fluorescent intensity of KS was calculated in lymphocytes (gate A) and microglia/macrophages (gate B) in the spinal cords. (**i**) KS expression in the gated lymphocytes and microglia/macrophages were calculated in normal and EAN spinal cords. (**j**) KS expression in the gated lymphocytes and macrophages were calculated in normal and EAN SNs. Open columns, normal rats; and closed columns, EAN rats. Each column represents average±S.D. The *P*-value was calculated using Student's two-tailed *t*-test. *P<0.05, **P<0.01. N.S., not significant

Immunohistochemical analysis revealed that microglia expressed KS but not CD68 in normal spinal cords (Figure 5a). In EAN, CD68 expression was induced and overlapped in Iba1-positive cells (Figure 5b). To further confirm the immunohistochemical findings, we enriched immune cells from the spinal cord. Immune cells purified from normal spinal cords using Percoll gradient expressed 5D4-reactive KSPGs, the molecular weights of which ranged 150–250 kDa on immunoblotting, and were consistent with those of original tissue lysate of normal spinal cords (Figure 6a, first and second lanes; compared with Figures 2 and 4). This KS expression was diminished at the peak of EAN (Figure 6a, third lane). Thus, these biochemical data were consistent with the immunohistochemical findings as shown in Figures 3 and 5.

We further investigated immune cells purified from the spinal cords by Percoll gradient. Most of the purified cells were negative of 7-amino-actinomycin D, suggesting that these are living cells (Figure 6b, upper panels). Using these living cells, flow cytometry analyses for CD11b, CD45, and KS were performed. In SNs, there were mainly two populations: CD11b-CD45+ cells (lymphocytes; Figure 6b, gate A) and CD11b+CD45+ cells (macrophages; Figure 6b, gate B). The latter dominantly contains CD11b+CD45high cells. Both the two populations increased in number in EAN (Figure 6b, lower panels; Figure 6c). There were also two main populations, i.e., CD11b-CD45+ lymphocytes (gate A) and CD11b+CD45+ microglia/macrophages (gate B) in the spinal cords, and both the two populations increased in number in EAN (Figure 6b, lower panels; Figure 6d). The CD11b+CD45+ cells in EAN spinal cords (gate B) may contain CD11b+CD45low microglia and CD11b+CD45high-activated microglia/peripheral macrophages. Indeed, CD45 expression was significantly increased in microglia/macrophages in EAN (Figures 6e and f).

We found that KS intensity was significantly decreased in CD11b+CD45+ microglia/macrophages in EAN spinal cords (Figures 6g and h). KS-positive microglia/macrophages were also strikingly reduced in number in EAN spinal cords (Figures 6g and i). In contrast, both CD11b-CD45+ lymphocytes (gate A) and CD11b+CD45+ macrophages (gate B) in SNs were KS negative (Figure 6j). Taken together, KS was specifically expressed on microglia/macrophages and disappeared in activated ones at the peak of EAN.

### Expression of pro-inflammatory cytokines are upregulated in the spinal cord of EAN

**Figure 7:**
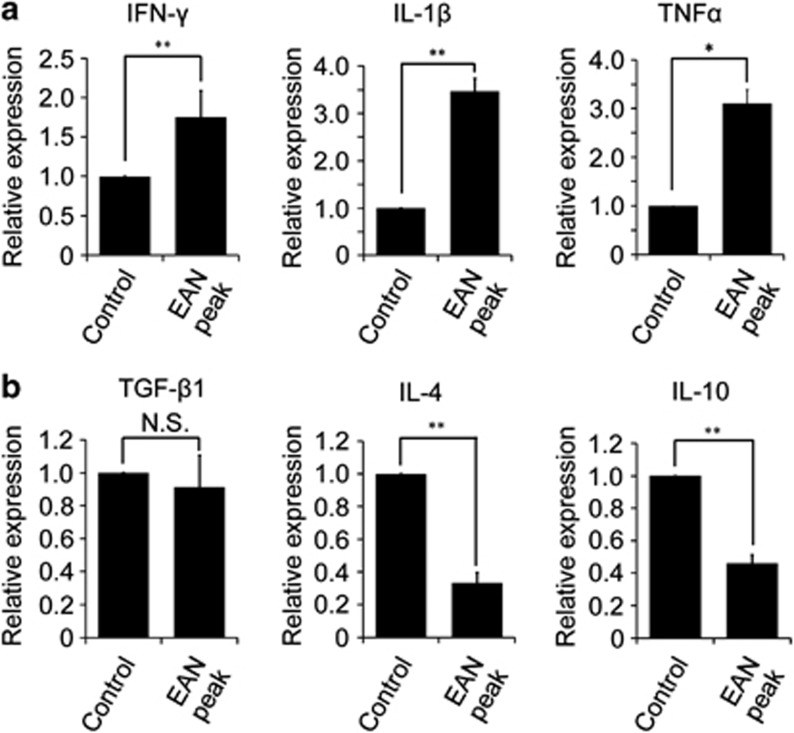
mRNA expression of pro-inflammatory and anti-inflammatory cytokines in the spinal cord. (**a**) Expression of pro-inflammatory cytokines. (**b**) Expression of anti-inflammatory cytokines. Each column represents average±S.D. The *P*-value was calculated using Student's two-tailed *t* test. *P<0.05, **P<0.01; *n*=3

The activation status of microglia/macrophages is influenced by pro-inflammatory and anti-inflammatory cytokines.^20^ We next asked what cytokines were produced in the spinal cord of EAN. We found that expression of pro-inflammatory cytokines, such as interleukin (IL)-1*β*, tumor necrosis factor (TNF)-*α*, and interferon (IFN)-*γ*, was increased in EAN (Figure 7a), whereas anti-inflammatory cytokines, including IL-4 and IL-10, were less expressed in EAN (Figure 7b).

### Expression of microglia/macrophages activation markers is upregulated by the knockdown of KSGal6ST

**Figure 8:**
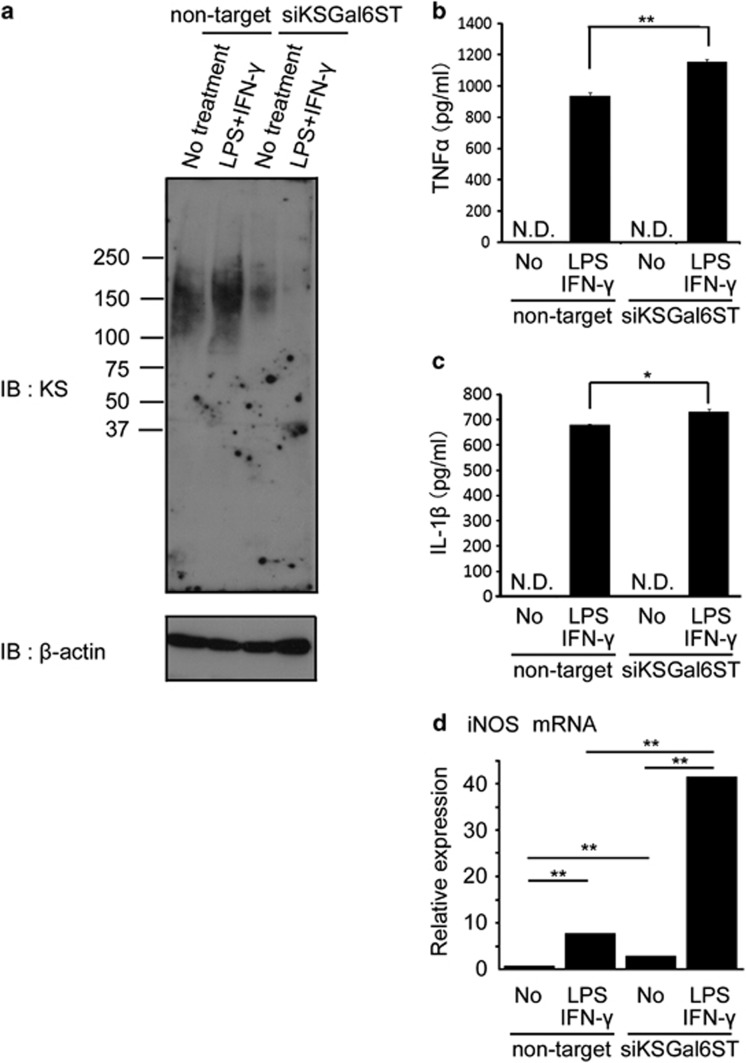
KSGal6ST knockdown attenuates the KS expression in primary cultured microglia and induces significantly higher expression of microglia/macrophages activation markers. (**a**) Total cell lysates of cultured microglia were subjected to immunoblotting with the anti-KS (5D4) and anti-*β* actin antibodies. The concentration of the inflammatory cytokines (TNF-*α* (**b**) and IL-1*β* (**c**)) in the culture media was measured by ELISA kit purchased from R&D Systems (Minneapolis, MN, USA). (**d**) mRNA expression of iNOS, a classically activated ‘M1' marker. Quantitative real-time PCR was performed to estimate mRNA expression. Each column represents average±S.D. The *P*-value was calculated using Student's two-tailed *t*-test. *P<0.05, **P<0.01; *n*=3

KS expression was observed in the primary cultured microglia. The M1-polarizing stimulus lipopolysaccharide (LPS)+IFN-*γ* alone could not diminish KS, rather appeared to upregulate KS. KSGal6ST is an essential enzyme for the biosynthesis of highly sulfated KS, which is recognized by the antibody 5D4. As expected, the 5D4-reactive epitopes were diminished after the knockdown of KSGal6ST under both non-treated and LPS/IFN-*γ*-stimulated conditions (Figure 8a). Importantly, the production of pro-inflammatory cytokines, such as TNF-*α* and IL-1*β*, was more upregulated by KSGal6ST knockdown under LPS/IFN-*γ* stimulation (Figures 8b and c). Furthermore, mRNA expression of the M1 marker iNOS was significantly upregulated after KSGal6ST knockdown (Figure 8d, columns 1 and 3). The expression of iNOS was elevated by the stimulation of LPS and IFN-*γ*, where KSGal6ST knockdown further accelerated the elevation (Figure 8d, columns 2 and 4). These *in vitro* expression profiles of markers were consistent with those observed *in vivo* as shown in Figures 4 and 7.

## Discussion

We revealed in this study the reverse relationship between KS expression and progression of EAN. Immunoblotting, immunohistochemistry, and flow cytometric analyses clearly demonstrated that KS expression in microglia/macrophages was lost from onset to remission stages of EAN. Although further studies are required to establish the usefulness of KS expression in human polyneuropathy, our study demonstrates that KS is a good marker for rat EAN.

We also found an interesting relationship of expressions of KS and microglia/macrophages activation markers. Microglia acquired CD68 expression, a general activation marker of microglia, during EAN, when KS expression was lost. It is obscure whether KS-positive cells switched to CD68-positive cells or KS-negative cells gave rise to CD68-positive cells. However, at least, the inflammatory milieu in the spinal cord in EAN may be important for expression changes of those markers. Thus, various inflammatory cytokines and chemokines are upregulated in the spinal cord after the onset of EAN.^20^ A previous report demonstrates that granulocyte/macrophage colony-stimulating factor or TNF-*α* increases KS expression in cultured microglia.^21^ If either factor is combined with IFN-*γ*, KS expression is strikingly suppressed.^21^ Indeed, we found that IFN-*γ* and TNF-*α* are both induced in the spinal cord of EAN (Figure 7). Interestingly, we also found that anti-inflammatory cytokines, i.e., IL-4 and IL-10 were suppressed in EAN. In this context, it is noteworthy that KS expression is enhanced in the spinal cord after SCI of rats and mice.^14, 15, 16^ KS expression is also induced in a cortical stab wound of mice and SOD1^G93A^ mice, a model of amyotrophic lateral sclerosis.^13, 22, 23^ Therefore, expression profiles and biological roles of KS may differ depending on inflammatory milieu in the CNS.

Regarding the role of KS in EAN, we performed experiments of KS knockdown in primary cultured microglia. We found that LPS+IFN-*γ* strikingly increased the production of TNF-*α* and IL-1*β*, as well as mRNA expression of iNOS, and that KSGal6ST knockdown enhanced these increases (Figure 8). Therefore, KS-less microglia were more sensitive to inflammatory stimuli; this is consistent with the *in vivo* findings of the association of EAN and diminished expression of KS in microglia/macrophages. However, further studies are required for the evaluation of the role of KS in microglia/macrophages in EAN. In addition, there is an alternative possibility that the diminished KS expression might be a consequence of inflammation. Thus, we previously revealed the upregulation of proteases in the injured spinal cord.^24, 25^ As the KSPGs are expressed on the cell surface of microglia in control rat, the upregulated proteases might degrade the core protein of KSPGs.

Although EAN is a representative model for autoimmune diseases of peripheral neurons, experimental autoimmune encephalomyelitis is a representative model for autoimmune diseases of the CNS and is a model for human multiple sclerosis. It has been reported that KS is downregulated in experimental autoimmune encephalomyelitis.^26, 27^ Furthermore, microRNA-124 promotes microglia quiescence and suppresses experimental autoimmune encephalomyelitis.^28^ MicroRNA-124 also downregulates pro-inflammatory cytokines and M1 markers (e.g., TNF-*α*, CD86, and nitric oxide) and upregulates anti-inflammatory cytokines and M2 markers (e.g., TGF-*β*1, arginase 1, and FIZZ1).^28^ Furthermore, it has been recently reported that various microRNAs including miR-27a, -29b, -125a, -146a -155, and -222 are implicated in polarized phenotypes of macrophages.^29^ Investigations on microRNAs in EAN might provide a clue to reveal the biological role of KS in this disease.

In summary, we found an interesting reverse relationship between KS expression and EAN pathogenesis, as well as microglial activation. Taken together previous reports of KS expression and functional recovery after neuronal injuries, our present study suggest that distinct inflammatory milieu in the CNS may determine expression profiles and biological roles of KS.

## Materials and Methods

### Animals and EAN induction

Male Lewis rats (ages: 8–10 weeks, 200–300 g; Japan SLC, Shizuoja, Japan) were housed under a 12-h light–12-h dark cycle with free access to food and water for 1 week. The institutional animal care and use committees of Nagoya University Graduate School of Medicine approved all experimental procedures. Rats were immunized in the hind footpad with 200 *μ*l of inoculum containing 100 *μ*g of synthetic P2 peptide (NH_2_-RTESTFKNTEISFKLGQEFEETTADN-COOH), representing amino acid residues 53–78 of human myelin P2 protein (TORAY, Tokyo, Japan). The peptide was dissolved in phosphate-buffered saline (PBS; 1 mg/ml) and then emulsified with an equal volume of CFA (Difco, Detroit, MI, USA) containing 1 mg/ml mycobacterium tuberculosis H37 Ra to get a final concentration of 0.5 mg/ml. Control rats were immunized with 200 *μ*l of inoculum containing PBS and CFA. Neurological symptoms of EAN were evaluated every day as follows: 0, normal; 1, limp tail; 2, abnormal gait; 3, mild paralysis; 4, moderate paralysis; and 5, complete paralysis.^30^ Control and EAN rats were perfused intracardially with 4% paraformaldehyde (PFA) followed by PBS under anesthesia, and the cervical, thoracic, thoraco–lumbar, lumbar spinal cords, and SNs were quickly isolated at 4, 7, 12, 18, 35, and 90 days after immunization. In order to evaluate the histological findings after the EAN induction, H&E and LFB staining was performed in those SNs. The paraffin-embedded sections were stained by LFB solution (0.1% LFB MBS and 0.05% acetic acid containing in 95% ethanol) overnight at 56 °C. After washing, the sections were briefly rinsed by 0.05% LiCO_3_ and 70% ethanol. After the rinsing with distilled water, the sections were incubated in Cresyl violet solution to stain the neurons for 6 min. After several washing, the sections were mounted with Eukitt (AS ONE, Osaka, Japan) and examined by an Olympus model BX41 microscope (Olympus, Melville, NY, USA).

### Western blotting

Spinal cords and SNs were homogenized in the lysis buffer (protease inhibitor cocktail (Nacalai Tesque, Kyoto, Japan) and 1% Triton X-100 in PBS) at 4 °C for 60 s. The homogenates were centrifuged at 20 000 × *g* for 15 min, and then the soluble protein concentrations were measured by Bradford protein assay using Protein Quantification Kit (Dojindo, Tokyo, Japan). The soluble proteins were treated with chondroitinase ABC (1 U/ml; Seikagaku Corporation, Tokyo, Japan) in 50 mM Tris-acetate buffer (pH 8.0) at 37 °C for 1 h and 20 *μ*l of 2 × SDS-PAGE sample buffer (0.125 M Tris-HCl, pH 6.8; 10% 2-mercaptoethanol; 4% SDS; 10% sucrose; 0.004% bromophenol blue) was added. The samples were subjected to SDS-PAGE after the boiling for 10 min. After transferring the proteins to a polyvinylidene difluoride membrane (Hybond-P; GE Healthcare, Uppsala, Sweden) and blocking it with PBS-T containing 5% skimmed milk, the membrane was blotted by mouse anti-KS antibody (1 : 1000; clone 5D4, Seikagaku Corporation), rabbit anti-Iba1 antibody (1 : 1000; Wako, Tokyo, Japan), mouse anti-CD68 anticody (1 : 1000; Millipore, Bedford, MA, USA), and goat anti-CD206 antibody (1 : 1000; R&D Systems). Anti-mouse IgG, anti-rabbit IgG, and anti-goat IgG conjugated with horseradish peroxidase (1 : 5000; Jackson Immunoresearch, West Grove, PA, USA) were used as secondary antibodies. Binding antibodies were visualized by ECL plus chemiluminescence system (GE Healthcare).

### Immunohistochemistry

Isolated spinal cords were fixed with 4% PFA in phosphate buffer (Wako) overnight, and cryoprotected by immersion in 20% sucrose in PBS during the subsequent night. The tissue samples were embedded in Tissue-Tek O.C.T. compound (Sakura Finetek, Tokyo, Japan) and quickly frozen by liquid nitrogen. Frozen tissues were cut into 20 μm sections on a cryostat (CM1800; Leica Instruments, Nussloch, Germany) and collected on MAS-coated glass slides (Superfrost; Matsunami Glass, Osaka, Japan). The sections were triple rinsed with PBS to wash out the cryoprotectant and fixed with 4% PFA in phosphate buffer, and permeabilized with PBS containing 0.1% Triton X-100. After blocking with 3% bovine serum albumin in PBS containing 0.1% Triton X-100, the sections were treated with chondrotinase ABC (0.5 U/ml) at 37 °C for 1 h, and then incubated with primary antibodies as follows; mouse anti-KS (1 : 200), rabbit anti-Iba1 for microglia/macrophages (1 : 200), mouse anti-CD68 for activated microglia/macrophages (1 : 200), and mouse anti-CD11b for microglia/macrophages (1 : 200; Biolegend, San Diego, CA, USA). After triple rinsing with PBS, the sections were incubated with secondary antibodies as follows; AlexaFluor 488-conjugated goat anti-mouse IgG, AlexaFluor 488-conjugated donkey anti-mouse IgG, AlexaFluor 594-conjugated goat anti-rabbit IgG, and AlexaFluor 594-conjugated donkey anti-goat IgG (1 : 500; Invitrogen, Paisley, UK). Subsequently, the sections were observed under a confocal microscopy A1Rsi (Nikon, Tokyo, Japan) and a fluorescent microscopy BZ-9000 (Keyence, Osaka, Japan).

### Cell preparation and flow cytometry

The animals were deeply anesthetized and the peripheral blood was completely removed by the perfusion with PBS. Flow cytometory of microglia/macrophages in the spinal cord and SNs was performed as previously described with minor modification.^31, 32, 33, 34^ Briefly, isolated spinal cords and SNs were carefully freed from meninges and mechanically dissociated in HBSS (Gibco, San Diego, CA, USA) containing 5 mg/ml collagenase (Wako). The mixture was incubated at 37 °C for 45 min, and the cell suspension was filtered through a 70-*μ*m cell strainer (Becton Dickinson, Heidelberg, Germany) into a new centrifuge tube. After rinsing twice with PBS, a part of cell pellet was resuspended with PBS and centrifuged through a 40%/70% Percoll gradient (stock isotonic Percoll was diluted with PBS) at 800 × *g* for 20 min. The cell pellet was rinsed twice by PBS and resuspended with FACS buffer (PBS containing 0.5% (w/v) bovine serum albumin and 2 mM EDTA). Fc*γ*R was blocked by the incubation with anti-CD32 antibody (1 : 400; BD Biosciences, San Jose, CA, USA) for 15 min at 4 °C. The prepared cells from the spinal cords and peritoneal cavity were stained with the following antibodies for 30 min at 4 °C; FITC-conjugated mouse anti-rat CD11b (eBioscience, Hatfield, UK), AlexaFluor 647-conjugated mouse anti-rat CD45 (Biolegend, Cambridge, UK), and anti-KS labeled using Zenon AlexaFluor 405 mouse IgG1 Labeling Kit (Invitrogen). After adding 300 *μ*l of FACS buffer and centrifuged at 5000 × *g* for 5 min, cell pellets were resuspended with 700 *μ*l of FACS buffer and were stained using 0.25 *μ*g/ml 7-amino-actinomycin (Sigma, St. Louis, MO, USA) for 10 min at room temperature. Cells were analyzed by FACS Canto2 3Laser-8color (Becton Dickinson) at the same parameters. The fluorescent compensation was performed using single-stained samples. The data were collected by analyzing 30 000 events using FACSDiva and FlowJo software (Becton Dickinson).

### Cell culture, siRNA transfection, and stimulation

Mixed glial culture was obtained from the postnatal day 1 rats purchased from Japan SLC. Primary cultured microglia was obtained by the shake-off method. Briefly, the flasks were shaken with 120 r.p.m. for 15 min at 37 °C. Floating microglia was cultured on the PLL-coated dishes in DMEM/F12 media supplemented with 10% FBS and antibiotics. One day later, the media were changed to the serum-free one and siRNAs were transfected using Lipofectamine RNAiMAX reagent (Invitrogen). One day later, microglia was stimulated by 1 *μ*g/ml LPS and 100 ng/ml IFN-*γ* (Peprotech, Rocky Hill, NJ, USA) for 24 h. Cells were collected and lysed by 1% Triton X-100 in PBS and lysates were subjected to SDS-PAGE.

### Real-time quantitative real-time PCR

RNA isolation was performed using RNeasy Lipid Tissue kit and RNeasy Mini kit (Qiagen, Valencia, CA, USA) according to the manufacturer's protocols. cDNA was prepared from 2 *μ*g of total RNA using an oligo(dT) primer and reverse transcriptase (Omniscript RT kit; Qiagen) following the standard protocols. Real-time PCR was performed using SYBR Green on the Mx3000P quantitative PCR System (Agilent Technologies, Santa Clara, CA, USA). Each PCR mixture contained 0.2 *μ*M of each primer, 10 *μ*l of 2 × SYBR Green Master Mix (Agilent Technologies), 3 *μ*l cDNA, 300 nM ROX reference dye, and PCR grade water (Roche Diagnostics, Mannheim, Germany) with a total volume of 20 *μ*l. The PCR was carried out with a denaturation step at 95 °C for 10 min and then for 50 cycles at 72 °C for 30 s and 60 °C for 1 min. All PCRs were performed in triplicate and normalized to internal control glyceraldehyde-3-phosphate dehydrogenase mRNA. Relative expression was presented using the 2^−(Ct experimental sample−Ct internal control sample)^ method. The sequence of primers was described in Table 1.

## Figures and Tables

**Table 1:** The primer sequences using quantitative real-time PCR of cytokines and activation markers

**Primers**	**Sequences**
*M1*	
IL-1*β*-f	5′-CACCTTCTTTTCCTTCATCTTTG-3′
IL-1*β*-r	5′-GTCGTTGCTTGTCTCTCCTTGTA-3′
TNF-*α*-f	5′-CCCAGACCCTCACACTCAGAT-3′
TNF-*α*-r	5′-TTGTCCCTTGAAGAGAACCTG-3′
IFN*γ*-f	5′-AGTCTGAAGAACTATTTTAACTCAAGTAGCAT-3′
IFN*γ*-r	5′-CTGGCTCTCAAGTATTTTCGTGTTAC-3′
iNOS-f	5′-CACAGTGTCGCTGGTTTGAA-3′
iNOS-r	5′-TCCGTGGGGCTTGTAGTTGA-3′
	
*M2*	
TGF-*β*1-f	5′-CGTGGAAATCAATGGGATCAG-3′
TGF-*β*1-r	5′-GGAAGGGTCGGTTCATGTCA-3′
IL-4-f	5′-GGTCACAGAAAAAGGGACTCCAT-3′
IL-4-r	5′-GCTCGTTCTCCGTGGTGTTC-3′
IL-10-f	5′-GCTCAGCACTGCTATGTTGC-3′
IL-10-r	5′-TTCATGGCCCTTGTAGACACC-3′
CD206-f	5′-TCAACTCTTGGACTCACGGC-3′
CD206-r	5′-ATGATCTGCGACTCCGACAC-3′

Abbreviations: IFN, interferon; IL, interleukin; TNF, tumor necrosis factor.

## Abbreviations

CFA: complete Freund's adjuvant
CNS: central nervous system
EAN: experimental autoimmune neuritis
IFN: interferon
IL: interleukin
KS: keratan sulfate
LFB: luxol fast blue
LPS: lipopolysaccharide
PBS: phosphate-buffered saline
PFA: paraformaldehyde
PG: proteoglycan
SCI: spinal cord injury
TNF: tumor necrosis factor
